# “Family shame should not be made public”: witnessing parental intimate partner violence and depressive symptom among Chinese college students —— the mediating role of brooding and the moderating role of resilience

**DOI:** 10.3389/fpsyg.2026.1904630

**Published:** 2026-09-30

**Authors:** Zijian Zhang, Weijin Li, Lihua Liu, Liping Wang, Ping Sun

**Affiliations:** 1 Department of School of Special Education and Rehabilitation, Shandong Medical and Pharmaceutical University - Yantai Campus, Yantai, China; 2 Department of Mood Disorders, Qingdao Mental Health Center, Qingdao, China

**Keywords:** brooding, Chinese college students, parental intimate partner violence witness, reflection, resilience

## Abstract

**Background:**

While Western research has consistently linked witnessing parental intimate partner violence (IPV) to elevated risks of depressive symptom among adolescents and young adults, the cognitive-emotional mechanisms underlying this association remain insufficiently understood within the Chinese cultural context. Witnessing parental IPV may represent not only a family-based traumatic experience but also a process through which enduring cognitive vulnerabilities become internalized within sociocultural environments. Although such culturally embedded schema-related vulnerabilities are not directly observable, they may be manifested through maladaptive cognitive processes such as brooding rumination, thereby increasing vulnerability to depressive symptom. From this perspective, the present study examined the mediating role of brooding in the association between parental intimate partner violence witness and depressive symptom, and the moderating role of resilience, thereby providing empirical insights into this culturally embedded schema-related vulnerability framework.

**Method:**

This cross-sectional questionnaire study used convenience sampling to recruit participants. The study investigated the mediating role of brooding (a maladaptive form of rumination) and the moderating role of resilience. Witness Parental IPV was measured by an adapted Chinese short-form of the Conflict Tactics Scale (CTS2), rumination was measured by the Ruminative Response Scale (RRS), resilience was measured by the Connor-Davidson Resilience Scale (CD-RISC), and depressive symptom measured by the Beck depressive symptom Inventory-II (BDI-II). A moderated parallel mediation model was conducted to test the proposed interrelationships among these variables.

**Results:**

This study involved 662 Chinese college students (44.6% male, aged 18 to 28 years, mean age = 21.66 years, standard deviation = 1.86). The findings indicate a positive correlation between the depressive symptom of these students and the level of witness parental IPV experienced. Notably, brooding, which is characterized as a maladaptive component of rumination, serves as a positive mediator in this relationship. In contrast, reflection, identified as an adaptive component of rumination, does not exhibit a similar association. Furthermore, resilience negatively moderates the relationship between brooding and depressive symptom, thereby being associated with a weaker association between brooding and depressive symptom.

**Conclusion:**

This study extends existing evidence on the association between parental intimate violence witnessing (IPV) and depressive symptom among Chinese college students. The findings suggest that brooding, rather than reflection, accounts for the association between parental IPV witness and depressive symptom, while resilience moderates the association between brooding and depressive symptom. These findings provide insight into the processes through which parental IPV witness may be associated with depressive symptom in the Chinese cultural context. The results highlight brooding and resilience as potential targets for interventions aimed at reducing depressive symptom among college students witnessing parental IPV.

## 1 Introduction

### 1.1 Witnessing IPV and depressive symptom

Depressive symptom is a prevalent mental health concern among college students (Zhang et al., 2025). Its manifestations include persistent feelings of sadness, diminished interest or pleasure in activities, and cognitive patterns that negatively influence an individual’s social and academic functioning (Tao et al., 2024). Depressive symptom has become a major mental health concern worldwide [World Health Organization (WHO), 2022], with a substantial burden among Chinese university students. A recent systematic review and meta-analysis reported that 34.7% of Chinese university students screened positive for depressive symptoms (Lin et al., 2025). Traumatic experiences serve as critical risk factors for the development of depressive symptom in this demographic, encompassing adverse childhood experiences such as emotional abuse, physical abuse, sexual abuse, emotional neglect, and physical neglect. These negative experiences can result in long-term dysregulation of an individual’s stress response system, thereby heightening the risk of subsequent psychological issues (Stewart et al., 2004). Prior studies have established a significant correlation between childhood trauma and the manifestation of depressive symptom in adulthood (Joshanloo et al., 2014). Notably, witnessing intimate partner violence (IPV) has emerged as a particularly salient early traumatic experience that has garnered increasing attention from researchers in recent years (Qin et al., 2024). Holden et al. defined witnessing parental IPV as being directly present and personally observing intimate partner violence occurring between one’s parents, rather than learning about the violence through others or other indirect sources. Witnesses do not participate in the conflict between their parents or experience the violence themselves; rather, they observe the occurrence of physical, psychological, and sexual violence between their parents (Holden, 2003). In China, approximately 30% of college students report having witnessed IPV between their parents (Fu et al., 2025).

Most studies examining the relationship between witnessing parental IPV and depressive symptom have been conducted in Western cultural contexts. Studies involving Western subjects consistently indicate that witnessing parental IPV heightens the risk of adverse psychological outcomes, including depressive symptom (Madruga et al., 2017; Hindin and Gultiano, 2006). However, individuals’ interpretations and coping mechanisms regarding witnessing domestic violence may vary significantly across different cultural backgrounds. In some Western cultural contexts, witnessing domestic violence may be perceived merely as a form of harm (Kulkarni et al., 2011). Conversely, witnessing parental IPV may not only be viewed as harmful but also imbued with the notion of family shame (Suh, 2020). An old Chinese saying goes, “family shame (called “Jia Chou” in Chinese) should not be made public,” reflecting this cultural belief. This saying underscores the idea that internal family conflicts and contradictions should remain private (Hou et al., 2021). In summary, Cultural differences may influence how individuals cognitively process traumatic experiences and regulate their emotions. Although witnessing parental IPV may be associated with negative psychological outcomes, such as depressive symptom, across both Chinese and Western cultural contexts (Jobson et al., 2022; Li et al., 2022), the cognitive and emotional processes underlying these associations may be shaped by specific cultural contexts. Therefore, it is necessary to further examine the psychological processes that may be involved in the association between parental IPV witness and depressive symptom within the Chinese cultural context. Therefore, this study aims to explore the relationship and potential psychological mechanisms linking witnessing parental IPV and depressive symptom among college students in China. The findings may offer targeted psychological intervention strategies and identify potential intervention targets for college students who have experienced parental IPV within the Chinese cultural framework.

### 1.2 The role of different rumination subtypes

Existing research primarily elucidates the mechanisms of rumination in the relationship between traumatic experiences and depressive symptom through the lenses of trauma theory, cognitive theory, and reaction style theory. Substantial evidence suggests that rumination serves as a significant mediator between traumatic experiences and depressive symptom (Kim et al., 2017; Deguchi et al., 2021). Nevertheless, investigations into the psychological mechanisms associated with witnessing parents’ intimate partner violence (IPV), a specific form of familial trauma, are relatively scarce. Considering that witnessing parents’ IPV constitutes a typical early adverse family experience, it is plausible to hypothesize that rumination may also be a crucial factor linking this experience to depressive symptom. Rumination is a cognitive processing style and emotion regulation strategy characterized by repetitive focus on one’s emotional state and its causes and consequences (Straus et al., 1998). Research indicates that exposure to violence is a significant predictor of rumination levels among college students (Chen et al., 2018). According to the cognitive model of rumination, negative external events, such as stressful life experiences and exposure to violence, can trigger ruminative thinking by heightening an individual’s attentional bias toward negative information (Zeng et al., 2015). Furthermore, rumination has been shown to significantly predict levels of depressive symptom and to elevate the risk of developing depressive symptom by prolonging and intensifying the processing of negative emotions (Kuyken et al., 2006; Nolen-Hoeksema, 1991; Roberts et al., 1998).

It is worth noting that, although numerous studies have explored the psychological mechanisms underlying the association between witnessing parental IPV and depressive symptom, few studies have utilized Early Maladaptive Schemas (EMS) theory as a framework to explain this relationship. According to Young et al. (2006) schema theory, Witnessing parental IPV may reflects exposure to persistent high-risk and unsafe conditions within an individual’s early family environment, such experiences have been theoretically linked to the emergence of maladaptive cognitive schemas, these schemas may, in turn, be associated with patterns of cognitive processing, including rumination, in response to stressful situations, thereby contributing to an increased vulnerability to depressive symptom (Balsamo et al., 2015). Furthermore, variations in family relational dynamics and norms regarding emotional expression across cultural settings may shape the development and expression of these schemas (Diržytė et al., 2024). Consequently, it is important to further examine the associations between parental IPV witness, rumination, and depressive symptom within the Chinese cultural context, in order to better understand cognitive internalization processes associated with early adverse family experiences.

Most studies have tended to present a negative and simplistic view of rumination. Research by Treynor et al. (2003) indicates that not all components of rumination are maladaptive. Rumination can be conceptualized as comprising two functional components: brooding and reflection. Brooding involves passive comparisons between one’s current circumstances and unattained goals, accompanied by repetitive focus on negative emotions, and is generally considered a maladaptive strategy for emotion regulation and coping. In contrast, reflection involves deliberate cognitive efforts to evaluate one’s situation and consider potential strategies for managing negative mood, representing a relatively adaptive form of rumination (Treynor et al., 2003).

Although the role of brooding in the association between negative life events and depressive symptom has received relatively consistent support, the function of reflection remains unclear, in particular, few studies have compared the differential roles of rumination subtypes in the association between witnessing parental IPV and depressive symptom among college students. A growing body of research has demonstrated that brooding consistently mediates the relationship between negative life events and adverse psychological outcomes. For example, brooding has been found to mediate the association between everyday discrimination and depressive symptom (Smith and Pössel, 2022), as well as the relationship between distress tolerance and posttraumatic stress symptoms (Lee et al., 2025). Similarly, among Chinese college students, Zeng et al. (2016) found that brooding mediated the association between childhood psychological maltreatment and trait depressive symptom. In contrast, the role of reflection in the relationship between negative life events and depressive symptom remains unclear, with previous findings yielding inconsistent and even contradictory results (Alıcı and Hasanlı, 2023).

Some studies have suggested that reflection may exhibit a risk pattern similar to brooding, with both subtypes showing significant associations with depressive symptom. These findings have been reported across different populations, including patients with depressive symptom (Chiang et al., 2022), college students (Alıcı and Hasanlı, 2023), and adults (Wang et al., 2023). However, other studies have proposed an opposing view,suggesting that reflection and brooding may serve distinct, or even opposite functions. Previous research has shown that the association between reflection and depressive symptom is weaker than that of brooding (Pedersen et al., 2022; Wei et al., 2024). Furthermore, a cross-cultural longitudinal study found that everyday discrimination was associated with subsequent depressive symptoms through brooding, whereas reflection did not show a significant association, suggesting that reflection may involve relatively more adaptive cognitive processes (Smith and Pössel, 2022). In addition, reflection has been associated with greater post-traumatic growth and better quality of life among patients with breast cancer, whereas brooding has not shown similar patterns (Tu, 2022).

In summary, the present study further compares the distinct roles of brooding and reflection in the association between witnessing parental IPV and depressive symptom among college students, aiming to clarify the functional differences between these two rumination subtypes.

### 1.3 The role of resilience

Previous studies investigating the mechanisms linking domestic violence and depressive symptom have primarily concentrated on risk factors, such as rumination, while neglecting the simultaneous consideration of other relevant psychological factors. Resilience, defined as an individual’s capacity to adapt actively and maintain mental health in the face of adversity, trauma, or significant stressors (American Psychological Association, 2010). Although excessive rumination is recognized as a significant cognitive vulnerability for depressive symptom, not all individuals exhibiting high levels of contemplative behavior develop severe depressive symptom. This observation suggests that other psychological factors modulate the relationship between rumination and the onset of depressive symptom. According to the resilience protection model, resilience functions as a psychological resource that may weaken the associations between risk factors and mental health outcomes (Askeland et al., 2020). Individuals with greater resilience are more likely to employ adaptive coping strategies, effectively regulate negative emotions, and maintain psychological equilibrium during stressful situations (Quan et al., 2017). In contrast, those with lower resilience are more vulnerable to the adverse emotions stemming from maladaptive cognitive processes. Previous research has demonstrated that resilience can attenuate the detrimental associations in the relationship between rumination and depressive symptom (Ji, 2024). Furthermore, individuals prone to rumination often experience heightened emotional distress, which may require additional psychological resources to manage. Such distress may be associated with an increased risk of developing depressive symptom (Lask et al., 2021). Resilience has been considered to reflect adaptive psychological resources available to individuals when coping with stress and adversity and has been associated with more effective emotion regulation and lower levels of rumination (Yu and Liu, 2025).

Therefore, psychological resilience may moderate the association between rumination and depressive symptom. Specifically, the positive relationship between rumination and depressive symptom is expected to be weaker among individuals with higher levels of resilience compared to those with lower levels of resilience.

### 1.4 The current study

The present study develops and tests a culturally embedded “culture–cognitive vulnerability” framework to explain the link between witnessing parental IPV and depressive symptom among Chinese college students. The theoretical contribution of this study is that integrating EMS theory with culturally specific family norms, positioning EMS as a key mechanism through which early family adversity is internalized and later expressed in cognitive–emotional dysfunction.

From a culturally embedded schema perspective,this study adopted a cross-sectional research design to examine: (a) whether witnessing parental IPV is associated with depressive symptom among Chinese college students; (b) whether brooding is expected to account for the association between parental IPV and depressive symptom, whereas reflection is not expected to show a significant indirect association and (c) whether resilience moderates the association between brooding and depressive symptoms, such that the relationship is weaker at higher levels of resilience.

## 2 Method

### 2.1 Procedure

After obtaining institutional approval from the Medical Ethics Committee of Shandong Medical And Pharmaceutical University (Approval Number: 2026-L032), this study was conducted in accordance with the ethical standards stipulated in the 1964 Declaration of Helsinki and its subsequent amendments or comparable ethical standards. The data was collected from online volunteers through convenient sampling in January 2026. Participants were informed of this study and guaranteed the anonymity and confidentiality of their responses. Then, the researchers created a questionnaire, with the informed consent form on the first page and the measurement results on the other pages. The link to the questionnaire was sent to individuals who voluntarily participated in the study. By now, all participants in this study have obtained informed consent forms. It will take about 10 min to implement these measures.

### 2.2 Participants

A total of 692 subjects were recruited, including undergraduate, master’s, and doctoral students. Participants were recruited from multiple universities across different regions of China, providing geographic diversity in the study sample. Inclusion criteria: Age 18 and above, current college enrollment, ability to complete questionnaires independently, voluntary participation, and signed informed consent. Thirty subjects were excluded due to invalid data. The specific exclusion criteria were as follows: (1) an abnormal questionnaire completion time, defined as a completion time of less than 120 s or more than 1,000 s; (2) an obvious mechanical response pattern, such as repeatedly selecting the same response option across a large number of consecutive items; and (3) failure on more than two attention-check items. Data finally analysis included 662 subjects (95.7% effective rate), with 44.56% male (*N* = 295) and 55.44% female (*N* = 367). The average age was 21.66 years (SD = 1.86).

### 2.3 Instrument

#### 2.3.1 Witnessing parent intimate partner violence

Intimate relationship violence (IPV) behaviors between parents were measured by using the Chinese short version of the Conflict Tactics Scale (CTS2) translated and revised by domestic scholars. This scale is widely used in the Chinese population and has been proven to have good reliability and validity (Pan et al., 2014). In the present study, the Chinese short form of the original CTS2 was appropriately adapted. Specifically, (1) corresponding IPV behavior items assessing “father-to-mother” and “mother-to-father” violence were combined to generate an indicator reflecting overall parental IPV exposure; and (2) the original response options assessing the specific frequency of IPV occurrence over the past year were simplified into a six-level frequency scale to improve the feasibility of retrospective reporting. These adaptations were based on the following considerations. First, similar adaptations of the CTS2 have been used in previous studies (Straus et al., 1996; Maguire-Jack et al., 2022; Cui et al., 2010; Babcock et al., 2019). Second, the present study primarily focused on the association between overall parental IPV exposure and depressive symptoms among college students; therefore, distinguishing the direction of violence was not necessary for the purpose of this study. Finally, as the participants were college students studying away from their family homes, accurately recalling the specific frequency of parental IPV during childhood may be difficult. Simplifying the frequency response options was therefore considered helpful for facilitating accurate retrospective reporting. The revised scale consists of 10 items, covering five dimensions: Negotiation, Psychological Aggression, Physical Assault and Sexual Coercion. All items are scored on a 6-point scale, from 1 = never to 6 = always,with questions 1 and 2 being scored in reverse. The higher the score, the more severe the IPV between parents. In this study, the Cronbach’s α coefficient of the revised CTS2 was 0.92, indicating that it has good internal consistency. Given that the CTS2 was adapted for the present study, we further conducted a confirmatory factor analysis (CFA) to examine its factor structure. The results indicated an overall acceptable model fit (χ^2^/df = 6.178, CFI = 0.972, TLI = 0.956, RMSEA = 0.089, 90% CI [0.076, 0.101], SRMR = 0.030), providing preliminary support for the factor structure of the adapted CTS2.

#### 2.3.2 Rumination

Use the Ruminative Responses Scale (RRS) to measure level of rumination, which was developed by Nolen-Hoesksema, translated and revised by Chinese scholars. This scale is widely used in China and has been proven to have good reliability and validity among Chinese subjects (Han and Yang, 2009). The scale contains 22 items such as “I often wonder why I always do this”, To avoid the high correlation issues caused by the overlap between the scale and the depressive symptom scale items, Treynor et al. removed 12 items that clearly overlapped with depressive symptoms, resulting in a two-dimensional scale comprising brooding and reflection. This scale has high internal consistency and test–retest reliability. This study adopted Treynor’s two-dimensional scale. Each item is rated on a 4-point scale ranging from 1 (never happens) to 4 (always happens), and questions 1 and 2 are reverse scored. The total score was calculated with higher score indicating higher level of rumination. In this study, the Cronbach’s α coefficient of RRS was 0.88. Among them, the Cronbach’s α coefficient of the brooding sub-scale was 0.81, and the Cronbach’s α coefficient of the reflection sub-scale was 0.80.

#### 2.3.3 Resilience

Use the Connor -Davidson Resilience Scale (CD-RISC) to measure resilience, which was developed by Connor and Davidson, translated and revised by Chinese scholars. This scale is widely used in China and has been proven to have good reliability and validity among Chinese subjects (Yu and Zhang, 2007). The scale contains 25 items such as “I can adapt to change”, including 3 sub-dimensions: resilience, strength and Optimism. Each item is rated on a 5-point scale ranging from 1 (never happens) to 5 (always happens). The total score was calculated with higher score indicating higher level of resilience. In this study, the Cronbach’s α coefficient of CD-RISC was 0.95.

#### 2.3.4 Depressive symptom

Use the Beck Depression Inventory-II (BDI-II) to measure depressive symptom, which was developed by Beck, translated and revised by Chinese scholars. This scale is widely used in China and has been proven to have good reliability and validity among Chinese subjects (Wang et al., 2011). The scale contains 21 items such as “Here are my feelings about sadness”, including 3 sub-dimensions: negative attitudes, physical symptoms, and executive function difficulties. Each item is rated on a 4-point, including four sentences describing feelings. The total score was calculated with higher score indicating higher level of depressive symptom. In this study, the Cronbach’s α coefficient of BDI was 0.95 (Table 1).

**Table 1:** Demographic variables. (*n* = 662).

Variables	Classification	Coding	*N* = 662	Percent
Gender	Male	1	295	44.6%
	Female	2	367	55.4%
Degree	Bachelor	1	593	89.6%
	Master	2	65	9.8%
	Doctor	3	4	0.6%
Birth Place	Rural	1	305	46.1%
	Urban	2	357	53.9%
Dad’s education	Below high school	1	216	32.6%
	High school	2	253	38.2%
	Bachelor	3	184	27.8%
	Master and above	4	9	1.4%
Mom’s education	Below high school	1	269	40.6%
	High school	2	222	33.5%
	Bachelor	3	166	25.1%
	Master and above	4	5	0.8%

### 2.4 Data analyses

All statistical analyses were conducted using IBM SPSS Statistics 26.0. First, to assess the potential impact of common method bias associated with the use of self-reported data, Harman’s single-factor test was conducted (Podsakoff et al., 2003). Second, independent-samples t tests and one-way analyses of variance (ANOVAs) were conducted to examine differences in the five core study variables across demographic characteristics. Third, based on previous research evidence, demographic variables, including gender, grade, place of origin, and parental educational level, were included as covariates in the subsequent analyses to control for potential demographic confounding factors associated with parental IPV witness, rumination, and depressive symptoms (Zhai et al., 2016). Fourth, correlations among the study variables were examined. Fifth, to test the research hypotheses, the original scores of parental IPV exposure, brooding, reflection, resilience, and depressive symptoms were converted into Z scores. PROCESS Model 4 (Hayes, 2017) was used to examine the parallel mediating roles of brooding and reflection in the association between witnessing parental IPV and depressive symptoms. Subsequently, to examine the moderating role of resilience in the association between brooding and depressive symptoms, an interaction term (brooding × resilience) was created by multiplying the standardized scores of brooding and resilience, and a moderated mediation model was tested using PROCESS Model 14 (Hayes, 2017). Before testing the research hypotheses, regression diagnostics were conducted for each regression equation to assess the assumptions of linearity, homoscedasticity, and multicollinearity, as well as to identify potential outliers and influential observations. All indirect and conditional indirect effects were estimated using 5,000 bootstrap resamples, and 95% CIs were calculated. An effect was considered statistically significant when its 95% CI did not include zero, and the significance level for regression coefficients was set at *p* < 0.05. The hypothesized model is presented in Figure 1.

**Figure 1:**
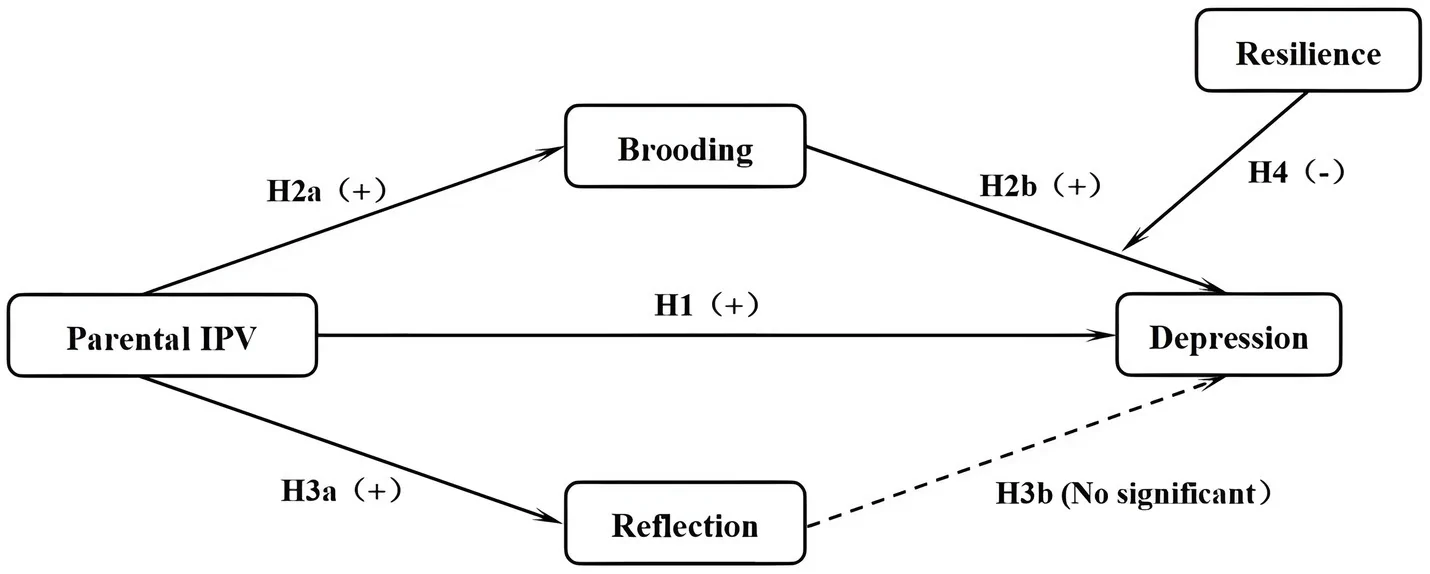
Hypothesized moderated mediation model.

## 3 Result

### 3.1 Common method bias test

The Harman’s single-factor test extracted seven factors with eigenvalues greater than 1 (without rotation), with the first factor accounting for 35.60% of the total variance, which was below the 40% reference threshold. These results suggest that common method bias may not be a primary source of variance in the present data; however, its presence cannot be completely ruled out based on this test alone. This limitation has been explicitly acknowledged in the Discussion, given that the study relied on self-reported data.

### 3.2 Differences in demographic variables and correlation analysis

The results indicated that all demographic variables showed significant differences across the core study variables, with detailed results presented in Table 2. Based on previous research and the results of preliminary analyses, partial correlation analyses were conducted to control for the potential confounding effects of demographic characteristics when examining the associations among variables. The results showed that witnessing parental IPV, brooding, reflection, depressive symptom, and resilience were significantly correlated with each other. Detailed correlation results are presented in Table 3.

**Table 2:** Differences in study variables across demographic characteristics (*n* = 662).

Demographic variable	IPV M ± SD	BRO M ± SD	RFL M ± SD	RES M ± SD	DEP M ± SD
Gender					
Male	17.96 ± 8.40	11.08 ± 3.30	11.31 ± 3.33	88.40 ± 16.94	33.16 ± 11.12
Female	18.00 ± 7.67	11.32 ± 3.11	11.27 ± 3.16	84.51 ± 18.39	34.74 ± 11.65
t	−0.07	−0.99	0.16	2.81^***^	−1.78
df	660				
*p*	0.95	0.33	0.87	0.01	0.08
Degree					
Bachelor	18.00 ± 8.08	11.34 ± 3.17	11.38 ± 3.21	86.45 ± 17.91	34.36 ± 11.45
Master	18.06 ± 7.46	10.22 ± 3.23	10.54 ± 3.45	83.95 ± 17.32	31.66 ± 11.16
Doctor	16 ± 3.92	8.75 ± 3.86	10.00 ± 2.83	93.75 ± 16.68	24.25 ± 1.71
F	0.13	4.88^**^	2.29	0.93	3.13^*^
df	2,659				
*p*	0.88	0.01	0.10	0.40	0.02
Post hoc		Bachelor vs. Mater			No significant
Birth place					
Rural	19.63 ± 8.71	11.80 ± 3.18	11.75 ± 3.18	83.30 ± 18.61	35.70 ± 11.92
Urban	16.57 ± 7.06	10.72 ± 3.14	10.89 ± 3.23	88.76 ± 16.79	32.62 ± 10.82
t	4.91^***^	4.39^***^	3.47^***^	−3.97^***^	3.46^***^
df	583.81	660	660	660	620.18
*p*	<0.001	<0.001	<0.001	<0.001	<0.001
Dad’s education					
Below high school	18.93 ± 8.29	11.75 ± 3.14	11.65 ± 3.05	83.96 ± 18.80	35.09 ± 11.63
High school	18.47 ± 8.17	11.03 ± 3.20	11.02 ± 3.28	85.30 ± 16.69	34.42 ± 11.33
Bachelor	16.27 ± 7.11	10.89 ± 3.21	11.26 ± 3.35	89.52 ± 17.75	32.47 ± 11.40
Master and above	16.67 ± 9.19	10.11 ± 2.93	10.67 ± 3.87	100.67 ± 13.53	29.89 ± 6.49
F	4.28^**^	3.37^*^	1.63	5.55^***^	2.26
df	3,658				
*p*	0.01	0.02	0.18	<0.001	0.08
Post hoc	Below high school vs. Bachelor high school vs. Bachelor	Below high school vs. high school		Below high school vs. Bachelor Below high school vs. Master and above	
Mom’s education					
Below high school	19.07 ± 8.42	11.62 ± 3.16	11.53 ± 3.18	84.45 ± 18.45	34.66 ± 11.04
High school	18.36 ± 7.93	10.91 ± 3.08	11.08 ± 3.22	84.91 ± 16.37	34.66 ± 11.83
Bachelor	15.64 ± 6.78	11.00 ± 3.34	11.20 ± 3.33	90.73 ± 17.98	32.22 ± 11.41
Master and above	20.20 ± 11.54	10.00 ± 4.06	9.80 ± 3.03	93.00 ± 21.67	33.40 ± 11.93
F	6.85^***^	2.64^*^	1.20	5.16^**^	1.90
df	3,658				
*p*	<0.001	0.05	0.31	<0.01	0.13
Post hoc	Below high school vs. Bachelor high school vs. Bachelor	No significant		Below high school vs. Bachelor high school vs. Bachelor	

IPV = Parental IPV; REF = Reflection; BRO = Brooding; DEP = Depression; RES = Resilience; M = mean; SD = standard deviation; t = t statistic; df = degrees of freedom; F = F statistic; Post hoc = results of pairwise post hoc comparisons; *p < 0.05; **p < 0.01; ***p < 0.001.

**Table 3:** Correlations among variables (*n* = 662).

Variables	*M*	*SD*	1	2	3	4	5
1. Parental IPV	17.98	8.00	1				
2. Brooding	11.21	3.20	0.34^***^	1			
3. Reflection	11.29	3.24	0.33^***^	0.73^***^	1		
4. Resilience	86.24	17.85	−0.52^***^	−0.29^***^	−0.15^***^	1	
5. Depression	34.04	11.43	0.60^***^	0.55^***^	0.46^***^	−0.63^***^	1

M = mean; SD = standard deviation; ^*^p < 0.05; ^**^p < 0.01; ^***^p < 0.001.

### 3.3 The roles of different rumination subtypes

The results showed that witnessing parental IPV was positively associated with depressive symptom (*β* = 0.47, *p* < 0.001). The total effect of witnessing parental IPV on depressive symptom was significant (total effect = 0.608), as was the direct effect after accounting for the two mediators (direct effect = 0.471, *SE* = 0.03, 95% *CI* [0.41, 0.53]). The total indirect effect through brooding and reflection was also significant (total indirect effect = 0.138). Furthermore, witnessing parental IPV was positively associated with brooding (*β* = 0.34, *p* < 0.001), and brooding was positively associated with depressive symptom (*β* = 0.37, *p* < 0.001). The indirect effect of witnessing parental IPV on depressive symptom through brooding was significant (indirect effect = 0.126, *SE* = 0.02, 95% *CI* [0.09, 0.17]), indicating a significant statistical indirect association. In contrast, witnessing parental IPV was also positively associated with reflection (*β* = 0.33, *p* < 0.001), whereas reflection was not significantly associated with depressive symptom (*β* = 0.03, *p* > 0.05). Consistent with this finding, the indirect effect through reflection was not significant (indirect effect = 0.012, *SE* = 0.01, 95% *CI* [−0.02, 0.04]). Thus, the significant total indirect effect was primarily attributable to the indirect association through brooding, whereas reflection did not significantly mediate the association between witnessing parental IPV and depressive symptom. Detailed results are presented in Table 4, and the standardized path coefficients of the parallel mediation model are illustrated in Figure 2.

**Table 4:** Parallel mediation effects of different rumination subtypes (*n* = 662).

Path	β	Effect	BootSE	t	*p*	BootLLCI	BootULCI	R^2^
Brooding								0.16
IPV → Brooding	0.34		0.04	9.39	< 0.001	0.27	0.42	
Reflection								0.12
IPV → Reflection	0.33		0.04	8.87	< 0.001	0.26	0.40	
Depression								0.51
IPV → depression	0.47		0.03	15.70	< 0.001	0.41	0.53	
Brooding → depression	0.37		0.04	8.89	< 0.001	0.29	0.45	
Resilience → depression	0.04		0.04	0.86	0.39	−0.05	0.12	
Total effect		0.47	0.03			0.55	0.67	
IPV → BRO → DEP		0.13	0.02			0.09	0.17	
IPV → REF → DEP		0.01	0.02			−0.02	0.04	
Total indirect		0.14	0.02			0.10	0.17	

IPV = parental intimate partner violence; REF = reflection; BRO = brooding; DEP = depression; RES = resilience; β = standardized regression coefficient; Effect = effect estimate; BootSE = bootstrap standard error; BootLLCI = lower limit of the 95% bootstrap confidence interval; BootULCI = upper limit of the 95% bootstrap confidence interval; t = t statistic; *p* = *p* value; R^2^ = proportion of variance explained by the regression model.

**Figure 2:**
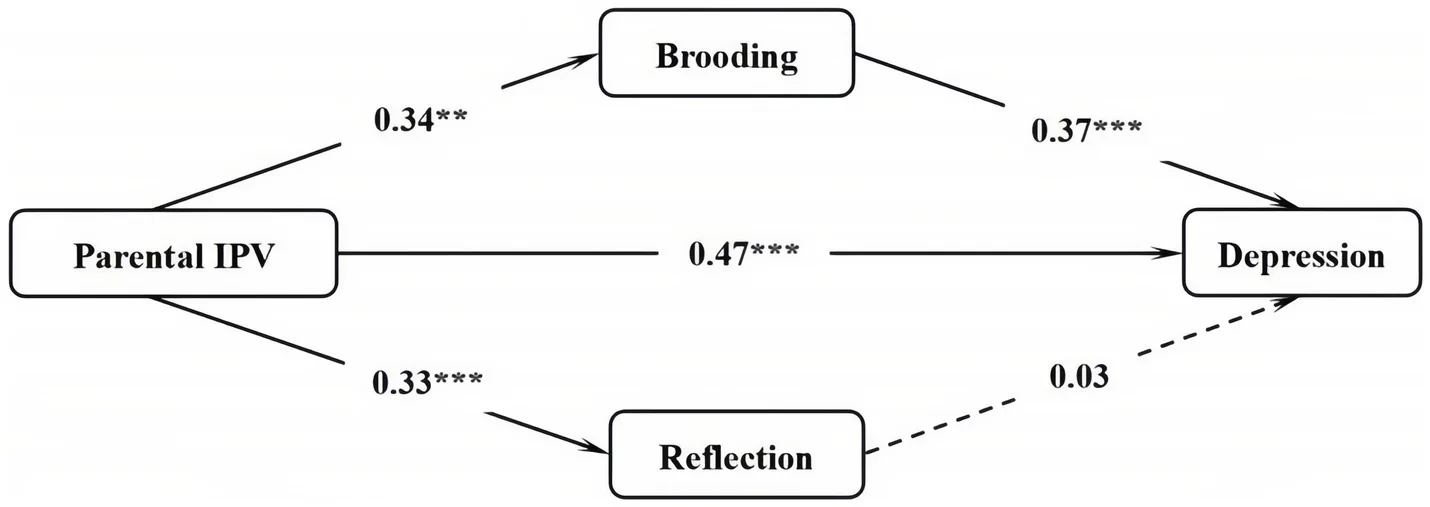
Parallel mediation model of brooding and reflection. *p < 0.05; **p < 0.01; ***p < 0.001.

### 3.4 The role of resilience

The results indicated that resilience significantly moderated the association between brooding and depressive symptom (*β* = −0.095, *p* < 0.001). Specifically, the positive association between brooding and depressive symptom was weaker at higher levels of resilience. The moderating effect of resilience on the association between brooding and depressive symptom is presented in Figure 3. To further examine whether resilience moderated the indirect association between witnessing parental IPV and depressive symptom through brooding, a moderated mediation analysis was conducted using PROCESS Model 14. As shown in Table 5, the direct effect of witnessing parental IPV on depressive symptom was significant (direct effect = 0.269). The conditional indirect effect of witnessing parental IPV on depressive symptom through brooding was significant at low, mean, and high levels of resilience, with effect estimates of 0.153 (95% CI [0.11, 0.19]), 0.120 (95% *CI* [0.09, 0.15]), and 0.088 (95% *CI* [0.06, 0.12]), respectively. The conditional indirect effect gradually decreased as resilience increased. Furthermore, the index of moderated mediation was significant (*index* = −0.03, 95% *CI* [−0.05, −0.01]), indicating that the indirect association between witnessing parental IPV and depressive symptom through brooding varied as a function of resilience. Specifically, the indirect association became weaker as resilience increased. The standardized path coefficients of the moderated mediation model are presented in Figure 4.

**Figure 3:**
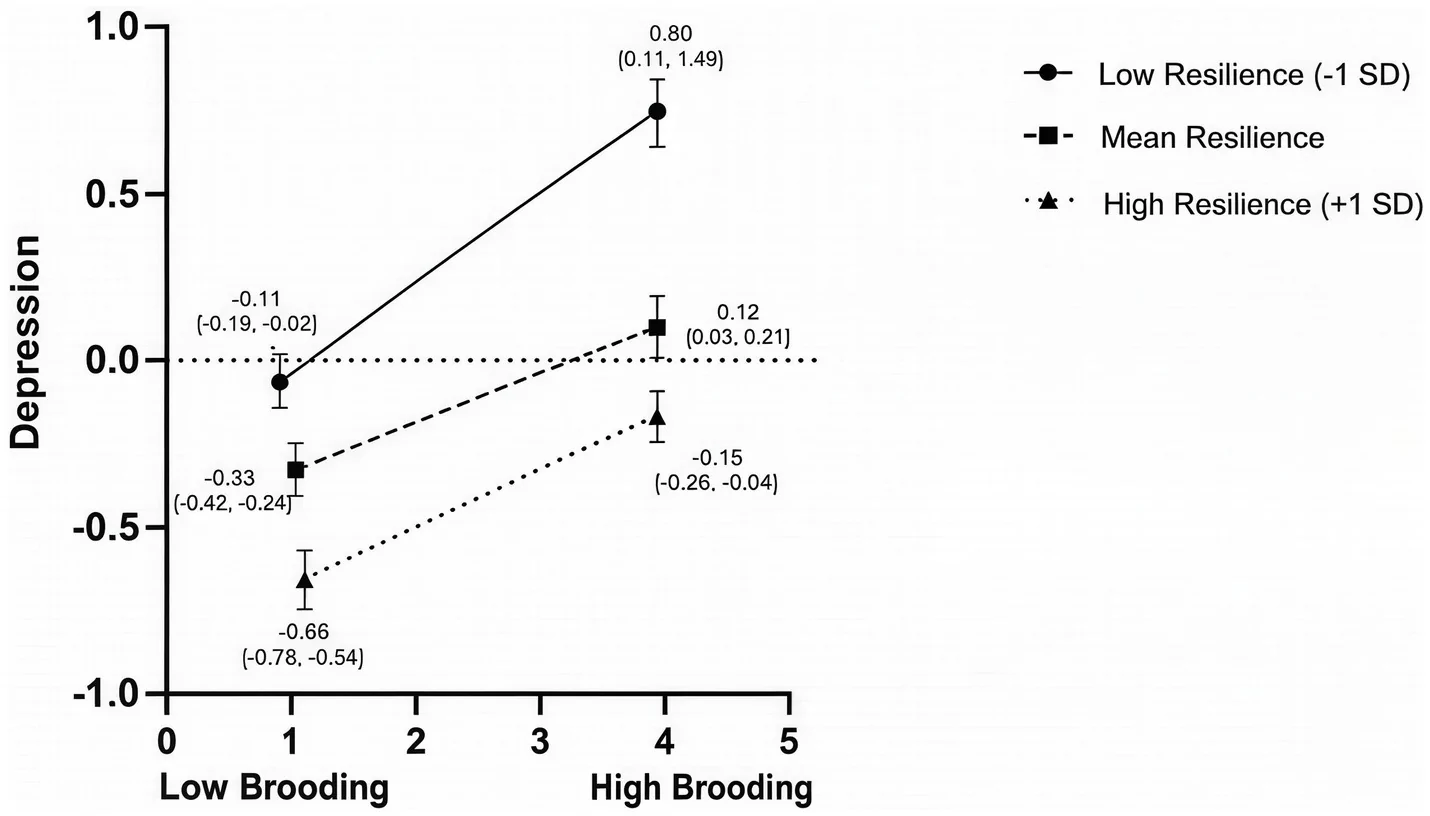
The moderating effect of resilience on the association between brooding and depression. Values are simple slopes of brooding predicting depression at low (1) and high (4) levels of brooding, at low (−1 SD), mean, and high (+1 SD) levels of resilience. Error bars represent 95% confidence intervals (CIs).

**Table 5:** Moderated effects of resilience (*n = 662*).

Path	β	Effect	BootSE	t	*p*	BootLLCI	BootULCI	R^2^	ΔR^2^
Brooding								0.16	
IPV → Brooding	0.34		0.04	9.38	< 0.001	0.27	0.42		
Depression								0.63	
IPV → depression	0.27		0.03	9.08	< 0.001	0.21	0.33		0.01
Brooding → depression	0.35		0.03	13.32	< 0.001	0.30	0.40		
Resilience →depression	−0.39		0.03	−13.41	< 0.001	−0.44	−0.33		
Brooding × Resilience → depression	−0.10		0.02	−4.47	< 0.001	−0.14	−0.05		
IPV → BRO → DEP									
Low resilience (−1 SD)		0.15	0.02			0.11	0.19		
Mean resilience		0.12	0.02			0.09	0.15		
High resilience (+1 SD)		0.09	0.01			0.06	0.12		
Index moderated mediation		−0.03	0.01			−0.05	0.02		

IPV = parental intimate partner violence; REF = reflection; BRO = brooding; DEP = depression; RES = resilience; β = standardized regression coefficient; Effect = effect estimate; BootSE = bootstrap standard error; BootLLCI = lower limit of the 95% bootstrap confidence interval; BootULCI = upper limit of the 95% bootstrap confidence interval; t = t statistic; *p* = *p* value; R^2^ = proportion of variance explained by the regression model.

**Figure 4:**
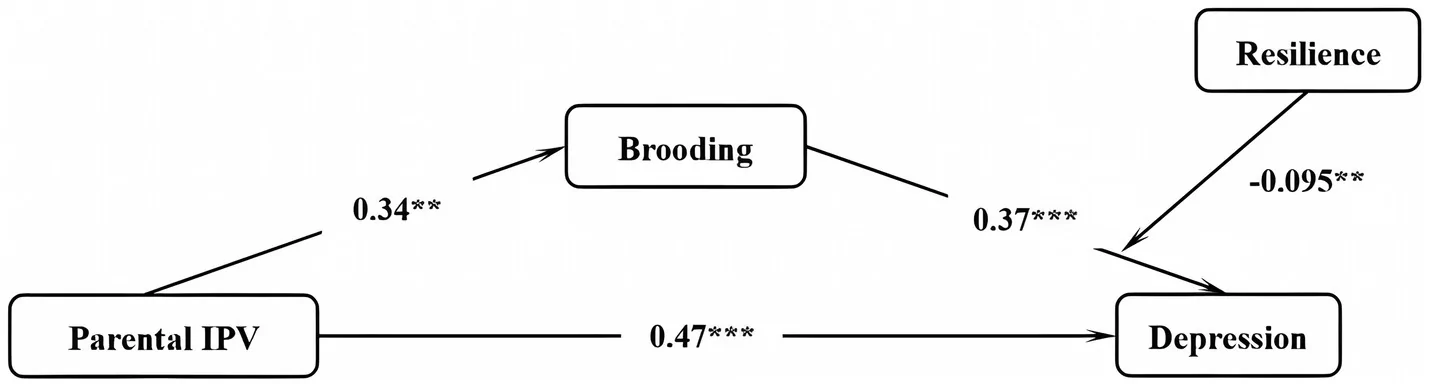
Moderated mediation model. *p < 0.05; **p < 0.01; ***p < 0.001.

## 4 Discussion

### 4.1 Witnessing parental IPV and depressive symptom

The present findings indicated a significant positive association between witnessing parental IPV and depressive symptom among Chinese college students, which is consistent with our hypothesis and with previous findings reported in Western cultural contexts (Menon et al., 2018; Stiller et al., 2022). Emotional Security Theory has frequently been used to interpret this association, suggesting that exposure to family violence and conflict may lead individuals to perceive the family environment as unstable and unpredictable. Such perceptions may undermine feelings of emotional security and be associated with the development of negative self-evaluations and maladaptive core beliefs, which could in turn contribute to elevated levels of depressive symptom (Davies and Cummings, 1994). However, although the adverse association between witnessing parental IPV and psychological well-being has been reported across both Western and Eastern cultural contexts, the mechanisms underlying this association may not be entirely identical across cultures (Davies et al., 2006). Family Systems Theory suggests that individual development is influenced not only by family-level interactions but also by broader cultural systems (Bowen, 1993). Accordingly, within the Chinese cultural context, witnessing parental IPV may be interpreted through culturally embedded beliefs and social norms, potentially shaping psychological responses in ways that differ from those proposed in predominantly Western frameworks.

In part of Western cultural contexts, witnessing parental IPV is may perceived primarily as a traumatic experience, which may prompt individuals to seek external support, such as counseling or social resources, to alleviate psychological distress (Singer et al., 1995; Chanmugam, 2015). However, within Chinese cultural contexts, for some individuals, parental conflict and an unstable family structure may be inconsistent with cultural norms that emphasize family harmony, and such inconsistencies could be related to adolescents’ psychological distress and experiences of cognitive dissonance (Markus and Kitayama, 2014). Furthermore, previous research has suggested that, in cultural contexts that emphasize collective relationships and family harmony, family conflict may give rise to negative self-evaluations, such as vicarious shame (Her and Joo et al., 2018; Wang et al., 2018). These cultural and psychological processes may provide a potential explanation for the association between witnessing parental IPV and depressive symptoms. In summary, the present findings provide a new perspective for understanding the psychological consequences of traumatic experiences within the Chinese cultural setting and may offer potential directions for future intervention research. It is important to note that the present study employed a cross-sectional questionnaire design and only examined the association between witnessing parental IPV and depressive symptom, without empirically testing the influence of the aforementioned cultural factors on this association. Therefore, the discussion of the cultural context in the present study mainly provides a new interpretive perspective that is consistent with the Chinese context, based on existing theories and research, rather than a direct empirical test of the relevant cultural mechanisms. Readers should interpret these findings with caution and avoid over-interpreting the cultural mechanisms involved.

### 4.2 The role of rumination

Firstly, witnessing parental IPV was positively associated with both brooding and reflection. Previous research has also suggested that adverse childhood experiences may increase individuals’ tendency to engage in persistent repetitive thinking and cognitive processing in response to later negative events (Deguchi et al., 2021; Hoffmann et al., 2024). Those research findings might be explained from the perspective of EMSs theory. EMS theory proposes that adverse early experiences may contribute to the development of persistent maladaptive cognitive patterns concerning the self, others, and the world. When individuals are repeatedly exposed to the conflict, instability, and unpredictability associated with parental IPV, such experiences may be related to the development or activation of maladaptive schemas involving insecurity, perceived threat, and mistrust of others. Activated EMSs may subsequently influence how individuals process negative experiences, making them more attentive to threatening or negative information and more likely to repeatedly think about adverse experiences and their potential causes (Young et al., 2006). Thus, witnessing parental IPV may be associated with greater rumination through such persistent negative cognitive processing. However, trauma-related repetitive cognitive processing may not necessarily manifest in a single form: Individuals may not only become passively immersed in the negative emotions elicited by traumatic experiences and their consequences, resulting in brooding characterized by maladaptive self-focused attention, but also repeatedly analyze the event from an objective third-person perspective in an attempt to understand the adverse experience, thereby facilitating active problem-solving efforts and reflecting a pattern of reflection (Treynor et al., 2003; Watkins and Roberts, 2020).

Secondly, the differences between rumination subtypes may be reflected more in their subsequent psychological adaptation outcomes rather than in whether they are elicited by traumatic experiences (Treynor et al., 2003; Zareian et al., 2021; Kim and Kang, 2022). The present study found that brooding seemed to show a stronger association with depressive symptom than reflection. Moreover, only brooding significantly predicted depressive symptom, whereas reflection did not. These findings suggest that different rumination subtypes may serve distinct psychological adaptation functions, with reflection showing lower maladaptive characteristics compared with brooding. This conclusion has also been supported by findings from various clinical populations. Among individuals with autism, brooding rather than reflection accounted for the association between autistic disorder and post-traumatic stress disorder (Golan et al., 2022). A longitudinal study among Taiwanese breast cancer patients further showed that brooding consistently predicted depressive symptom two years later, whereas reflection did not exhibit the same association (Wang et al., 2021). Beyond psychological evidence, neuroimaging findings have also provided evidence for the possibility that different rumination subtypes may involve distinct psychological adaptation functions. For example, Wang et al. (2022) found that brooding and reflection exhibited distinct patterns of resting-state electroencephalographic dynamics. Specifically, brooding showed a closer association with abnormal neural activity patterns related to major depressive disorder, whereas reflection did not demonstrate a similar neural association. These empirical findings can be interpreted from the perspective of response styles theory. This theory proposes that brooding involves passively comparing one’s current situation with unattained desired standards, which may lead attention to remain focused on one’s negative emotional states and their consequences. In contrast, reflection represents a more purposeful form of self-focused cognitive processing, through which individuals analyze and understand problems in an attempt to identify potential solutions (Nolen-Hoeksema et al., 2008).

In summary, following witnessing parental IPV, brooding may keep individuals engaged in passive processing of traumatic experiences, negative emotions, and their consequences, thereby potentially increasing vulnerability to depressive symptom. Although reflection may also be activated by traumatic experiences, its relatively more purposeful and problem-solving-oriented characteristics may prevent it from showing the same association with depressive symptom risk as brooding. Therefore, when future research examines the role of rumination in the association between witnessing parental IPV and depressive symptom, rumination should not be conceptualized as a single and homogeneous maladaptive cognitive process. Instead, attention should be given to the distinct functions of different rumination subtypes and their differential associations with psychological health outcomes.

### 4.3 The moderating role of resilience

The present study found that resilience attenuate the association between brooding and depressive symptom. Specifically, the association between brooding and depressive symptom was weaker among individuals with higher levels of resilience, whereas this association was stronger among individuals with lower levels of resilience. This finding was consistent with our hypothesis and previous empirical evidence. A study conducted among Chinese student athletes during the COVID-19 pandemic found that resilience was significantly negatively associated with rumination, depressive symptom, and self-harm behaviors. Meanwhile, resilience may provide individuals experiencing adversity with psychological adjustment and recovery resources, which may finally reduce repetitive negative thinking, thereby attenuating the adverse association between rumination and self-harm behaviors (Zhao et al., 2023). Similarly, a study among adolescents with depressive symptom indicated that resilience could attenuate the association between rumination and self-harm behaviors, suggesting that enhancing resilience may represent a potential approach for the prevention and intervention of self-harm behaviors (Wang et al., 2025). Ursu and Măirean (2022) further found that higher levels of resilience were associated with less frequent use of maladaptive strategies for managing negative emotions, such as self-blame, catastrophizing, and repetitive thinking (Ursu and Măirean, 2022). Individuals with greater resilience were more likely to employ cognitively adaptive emotion regulation strategies and exhibit better psychological well-being. Collectively, higher resilience may be associated with a weaker association between brooding associated with witnessing parental IPV and depressive symptom (Fergus and Zimmerman, 2005). Therefore, resilience may moderate the association between depressive symptom and brooding that is associated with witnessing parental IPV. Individuals with higher levels of resilience may activate more positive factor, which can interact with threat-related factors arising from traumatic experiences, alleviate the disruptive associations of brooding with psychological well-being, and ultimately reduce depressive symptom (Fergus and Zimmerman, 2005). Furthermore, previous research has suggested that individuals may acquire positive psychological resources to enhance resilience across different developmental stages, thereby facilitating positive adaptation and personal growth. Resilience may reduce individuals’ reliance on brooding by strengthening their sense of autonomy and enhancing self-understanding, encouraging them to adopt more positive and adaptive coping strategies (Abulfaraj et al., 2024; Rosenbaum et al., 2022). Taken together, the present study revealed the joint roles of the risk factor (brooding) and resilience in the association between witnessing parental IPV and depressive symptom among college students. Specifically, brooding represents an important cognitive risk factor, whereas resilience moderates the latter part of this risk association. In practice, interventions aimed at reducing brooding tendencies and enhancing resilience may provide a potential intervention approach for reducing the incidence of depressive symptom among college students who have experienced parental IPV.

### 4.4 Limitations and future research

First, the introduction of Chinese culture-related factors and early maladaptive schemas (EMSs) was intended to provide background information and a theoretical framework for interpreting the findings, rather than to include them as core variables for measurement and empirical testing. Therefore, the findings should be interpreted with caution, and inferences regarding the roles of Chinese cultural factors or EMSs should not extend beyond what is supported by the data. The conclusions supported by the present data are primarily limited to the statistical associations among parental IPV exposure, depressive symptoms, brooding, reflection, and resilience. Future research could incorporate Chinese culture-related factors (e.g., face culture, family shame culture, and patriarchal culture) and EMSs as core variables and directly measure and empirically examine them, thereby providing stronger evidence for interpreting the relationships among these variables from the perspectives of Chinese culture and EMSs theory.

Second, the cross-sectional design of the present study prevents the determination of the temporal ordering among parental IPV exposure, rumination, resilience, and depressive symptoms, and therefore precludes causal inferences. Although our findings are consistent with the proposed mediation and moderation mechanisms, they only reflect statistical associations among the variables and cannot establish that parental IPV exposure leads to rumination or depressive symptoms, or determine the temporal roles of rumination and resilience. Therefore, these findings should be interpreted with caution. Future research should employ longitudinal designs with multiple waves of assessment to establish the temporal ordering of these variables, examine their potential causal relationships, and provide stronger evidence for the proposed mediating and moderating roles of rumination and resilience.

Third, the CTS2 was appropriately adapted in the present study. Although the adapted measure demonstrated high internal consistency and received preliminary support for its factor structure through confirmatory factor analysis (CFA), these findings cannot completely rule out potential measurement biases introduced by the adaptations. Specifically, combining corresponding IPV items for both parents may have resulted in the loss of information regarding the direction of violence and certain specific violent behaviors, while simplifying the original frequency response options may have reduced sensitivity to differences in the actual frequency of IPV exposure, thereby limiting the precision with which individual differences in IPV exposure could be assessed. Therefore, the findings should be interpreted with caution. Future research should further validate the measurement properties of the CTS2 in Chinese samples while retaining its original measurement structure and frequency information where feasible, and examine differences across the direction and types of IPV.

Fourth, the present study could not adequately distinguish among direct maltreatment, involvement in parental IPV conflicts, and witnessing parental IPV as three distinct forms of violence exposure, nor could it rule out the possibility that these experiences co-occurred within the same participants. Therefore, the observed associations between witnessing parental IPV, rumination, and depressive symptoms may have been influenced by other forms of maltreatment, making it difficult to determine whether the psychological outcomes could be specifically attributed to witnessing parental IPV. Future research should more carefully distinguish and assess different forms of maltreatment and violence exposure and compare their relative associations with psychological outcomes to further clarify the unique role of witnessing parental IPV.

Fifth, confirmatory factor analysis (CFA) was not conducted for the three measures of depressive symptoms (BDI-II), psychological resilience (CD-RISC), and rumination (RRS). Therefore, fit indices such as CFI, TLI, and RMSEA were not available, and their factor structures and measurement validity could not be fully evaluated in the present sample. In addition, the present study used total scale scores as observed variables in the regression models and did not fully account for measurement error, which may have affected the estimation of the associations among the variables. Future research should further use CFA to validate the factor structures and measurement validity of these measures in Chinese samples and, where feasible, employ latent variable models to better account for measurement error.

Sixth, because the present study primarily relied on retrospective self-reports, the potential influence of common method bias on the findings cannot be completely ruled out. Although Harman’s single-factor test was used to provide a preliminary assessment of common method bias, this approach has inherent limitations, and its results cannot demonstrate that common method bias is absent. Therefore, the findings should be interpreted with caution. Future research could adopt multi-source and multi-method approaches, such as incorporating reports from parents or other family members, objective data, and longitudinal assessments, to reduce the common method bias associated with reliance on a single self-report method and further strengthen the robustness of the findings.

Finally, the participants in the present study were drawn from a convenience sample, primarily consisting of Chinese university students recruited via online platforms. The homogeneity of the university student population in terms of age, educational background, and life experience suggests that caution should be exercised when generalizing findings to non-student populations or individuals at different developmental stages. Future research could adopt stratified random sampling or recruit participants across multiple regions and populations to enhance external validity. Combining online surveys with offline interviews or employing multi-method assessment designs may also help reduce the limitations associated with single self-report measures and further examine the applicability and stability of the findings across diverse cultural and developmental contexts.

## 5 Conclusion

To examine the associations between witnessing parental IPV and depression and the potential roles of brooding, reflection, and resilience among college students, the present study employed a moderated mediation model. The findings indicated that, first, witnessing parental IPV was significantly associated with depression, brooding, reflection, and resilience, with significant correlations observed between every pair of these variables. Furthermore, witnessing parental IPV was associated with depression both directly and indirectly through brooding, whereas reflection did not mediate this association. Finally, resilience negatively moderated the association between brooding and depression, such that the association was weaker among individuals with higher levels of resilience than among those with lower levels of resilience. The present study extends our understanding of the association between witnessing parental IPV and depression among Chinese college students and provides further evidence for the differential roles of different rumination subtypes. The findings also identify potential targets for interventions for individuals with experiences of witnessing parental IPV. Specifically, interventions may benefit from targeting reductions in brooding while strengthening resilience, which may help address the adverse psychological consequences associated with traumatic experiences and potentially reduce depressive symptoms while promoting psychological well-being among college students.

## Data Availability

The raw data supporting the conclusions of this article will be made available by the authors, without undue reservation.
