# Supplementary material for: “Family shame should not be made public”: witnessing parental intimate partner violence and depressive symptom among Chinese college students —— the mediating role of brooding and the moderating role of resilience
Source: Front Psychol. 2026 Sep 30;17:1904630. doi: 10.3389/fpsyg.2026.1904630 (PMC13661552; doi:10.3389/fpsyg.2026.1904630)
Supplement: Supplementary file 1 [file Data_Sheet_1.pdf]

1 Description of the Adapted Chinese Short Form CTS2

1.1 Specific Items

1. My father and mother were able to explain themselves to each other and compromise.

2. My father and mother were able to express care and respect toward each other.

3. My father insulted or verbally abused my mother (my mother insulted or verbally abused my father).

4. My father pushed or slapped my mother (my mother pushed or slapped my father).

5. My father threw something at my mother or threatened to hit her (my mother threw something at my father or threatened to hit him).

6. My father punched or kicked my mother (my mother punched or kicked my father).

7. My father and mother had a physical fight in which my father injured my mother (my father and mother had a physical fight in which my mother injured my father).

8. My father and mother had a physical fight that resulted in one of them being hospitalized.

9. My father forced my mother to have sexual intercourse with him (my mother forced my father to have sexual intercourse with her).

10. My father forced my mother to have sexual intercourse with him without using protection (my mother forced my father to have sexual intercourse with her without using protection).

1.2 Response Options and Scoring of the Adapted Version

In the original CTS-2 version, participants responded based on the exact number of times each behavior had occurred within the past year; the specific response options and their corresponding codes were as follows: 0 = Never; 1 = Once; 2 = Twice; 3 = 3 – 5 times; 4 = 6 – 10 times; 5 = 11 – 20 times; 6 = 21 – 50 times; 7 = More than 50 times. According to the scoring methodology of the original CTS-2, a higher score indicates a higher frequency of occurrence for the corresponding behavior. The Chinese version of the CTS-2 Short Form used a six-level frequency scale for assessment: 1 = Never; 2 = Rarely; 3 = Occasionally; 4 = Sometimes; 5 = Frequently; 6 = Always. Each item in the adapted version was scored using this six-level scale; a higher score indicates a higher frequency of occurrence for the corresponding behavior.

1.3 Classification of Dimensions

Negotiation：1、2；Psychological Aggression：3、5；Physical Assault：4、6、7、8

Sexual Coercion：9、10

2 Descriptive Statistics for Parental IPV Witness Scores

2.1 descriptive statistics index

**Statistics**	
N	662 *(no missing data)*
M	17.98
SD	8.00
Median	15.00
Mode	11.00
Mix	10.00
Max	53.00
Skewness	1.66
Kurtosis	2.65

2.2 Different types of IPV exposure among parents

An analysis of the current level of parental IPV exposure among the university student sample revealed that a total of 232 participants reported having experienced parental IPV exposure, accounting for 35% of the total sample. Overall, the mean score for parental IPV exposure was 17.98 ± 8.00, with scores ranging from 10 to 60. Based on the CTS-2 scoring criteria, the degree of parental IPV exposure among the university students was classified according to the occurrence of different types of intimate partner violence perpetrated by parents. Specifically: if the score for all items was ⩽2, the individual was classified as having no parental IPV exposure; if at least one item on the psychological aggression dimension scored ⩾3, the individual was classified as having mild parental IPV exposure; if at least one item on the physical aggression dimension scored ⩾3, the individual was classified as having moderate parental IPV exposure; and if at least one item on the sexual coercion dimension scored ⩾3, the individual was classified as having severe parental IPV exposure. The study findings indicated that 100 participants (15%) experienced mild parental IPV exposure; 38 participants (6%) experienced moderate parental IPV exposure; and 94 participants (14%) experienced severe parental IPV exposure. Additionally, the descriptive statistics for each dimension of the CTS-2 scale (negotiation, psychological aggression, physical aggression, and sexual coercion) are provided in **Table 1.**

Table 1

Descriptive Statistics for the Dimensions of the CTS2 *(n = 662)*

Item	*n*	*M*	*SD*	*Max*	*Min*
Parental IPV witness	662	17.98	8.00	53	10
Negotiation	662	5.47	2.43	12	2
Psychological Aggression	662	3.65	1.96	12	2
Physical Assault	662	6.09	3.45	24	4
Sexual Coercion	662	2.77	1.63	12	2

2.3 Ceiling effect and floor effect analysis

**Index**	**Result**
Theoretical range	10 – 60
Observed range	10 – 37
Floor effect	6.0%
Ceiling effect	0%

*The adapted CTS2 scores ranged from 10 to 37, with a theoretical range of 10 – 60. Among the participants, 6.0% scored at the theoretical minimum, whereas none scored at the theoretical maximum, indicating no substantial ceiling effect and a relatively limited floor effect

2.4 Diagnostic Results for Outliers and Influential Observations

**Diagnostic indicator**	**Range**	**Criterion**	**Interpretation**
Standardized residual	[−2.783, 2.902]	|residual| ≤ 3	No obvious outliers
Studentized deleted residual	[−2.842, 2.922]	|residual| ≤ 3	No obvious outliers
Cook's distance	[0.000, 0.126]	< 1	No influential observations
Centered leverage value	[0.000, 0.029]		No obvious high-leverage observations

2.5 McDonald's ω:

we used IBM SPSS Statistics 26.0 and calculated the McDonald's ω coefficient using the **OMEGA extension**. Ten items from the adapted CTS2 were included as observed measures in the analysis; the results yielded a McDonald's ω =.914.

3 Results of Linearity, Multicollinearity, and Homoscedasticity Diagnostics

3.1 Equation 1: IPV + covariates → brooding

- **scatter plot**

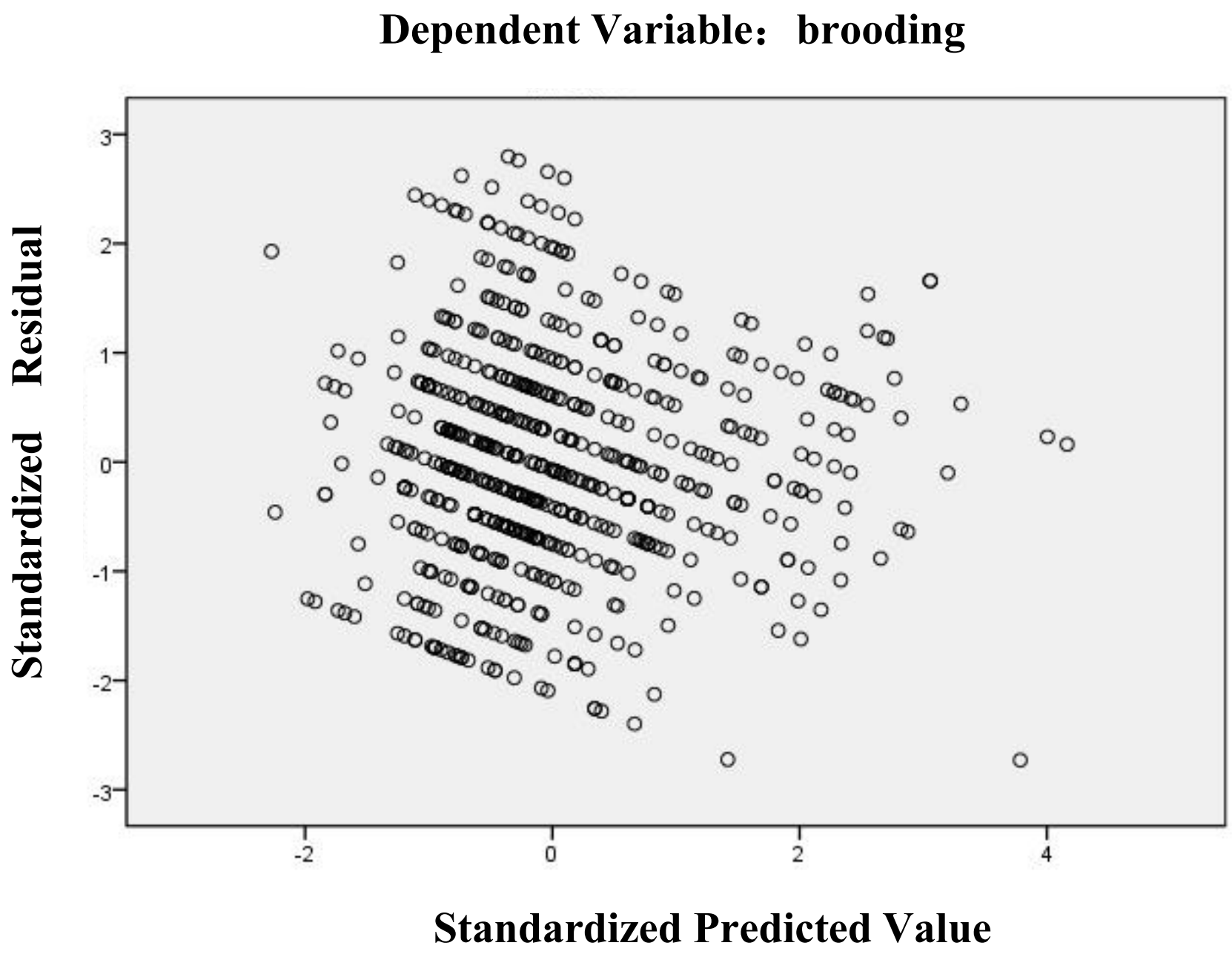

Table 2-1

Regression Coefficients and Multicollinearity Diagnostics

Variable	Unstandardized Coefficients		standardized Coefficients			Collinearity Statistics	
	b	*SE*	*β*	*t*	*Sig.*	*Tolerance*	*VIF*
Constant	10.725	0.754		14.226	＜0.001		
gender	0.204	0.233	0.032	0.876	0.382	0.977	1.024
El	-1.065	0.346	-0.111	-3.075	0.002	0.991	1.009
Bp	-0.620	0.254	-0.097	-2.446	0.015	0.820	1.219
Fe	-0.313	0.229	-0.079	-1.363	0.173	0.379	2.641
Me	0.247	0.230	0.063	1.074	0.283	0.372	2.686
IPV	0.137	0.015	0.344	9.377	＜0.001	0.956	1.046

Note. *El = Education level; Bp = Birthplace; Fe = Father's Education; Me = Mother's Education; IPV = Intimate Partner Violence witness. Blow as the same.*

3.2. Equation 2: IPV + covariates → reflection

- **scatter plot**

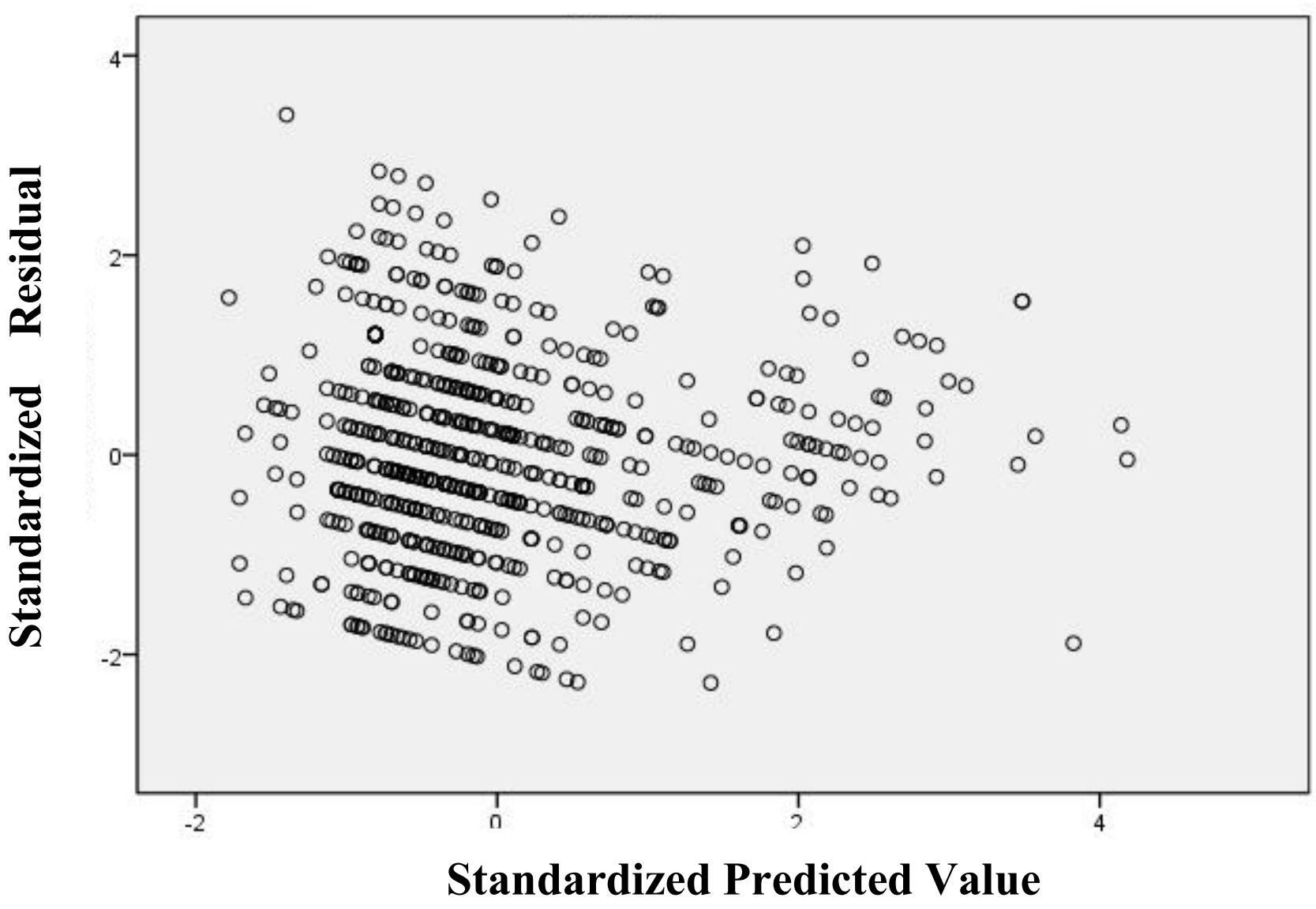

Table 2-2

Regression Coefficients and Multicollinearity Diagnostics

Variable	Unstandardized Coefficients		standardized Coefficients			Collinearity Statistics	
	b	*SE*	*β*	*t*	*Sig.*	*Tolerance*	*VIF*
Constant	10.408	0.776		13.407	＜0.001		
gender	-0.048	0.240	-0.007	-0.201	.841	0.977	1.024
El	-0.768	0.357	-0.079	-2.153	.032	0.991	1.009
Bp	-0.500	0.261	-0.077	-1.915	.056	0.820	1.219
Fe	-0.042	0.236	-0.011	-0.179	.858	0.379	2.641
Me	0.136	0.237	0.034	0.575	.565	0.372	2.686
IPV	0.134	0.015	0.331	8.868	＜0.001	0.956	1.046

3.3 Equation 3: IPV + brooding + reflection+ covariates → depression

- **scatter plot**

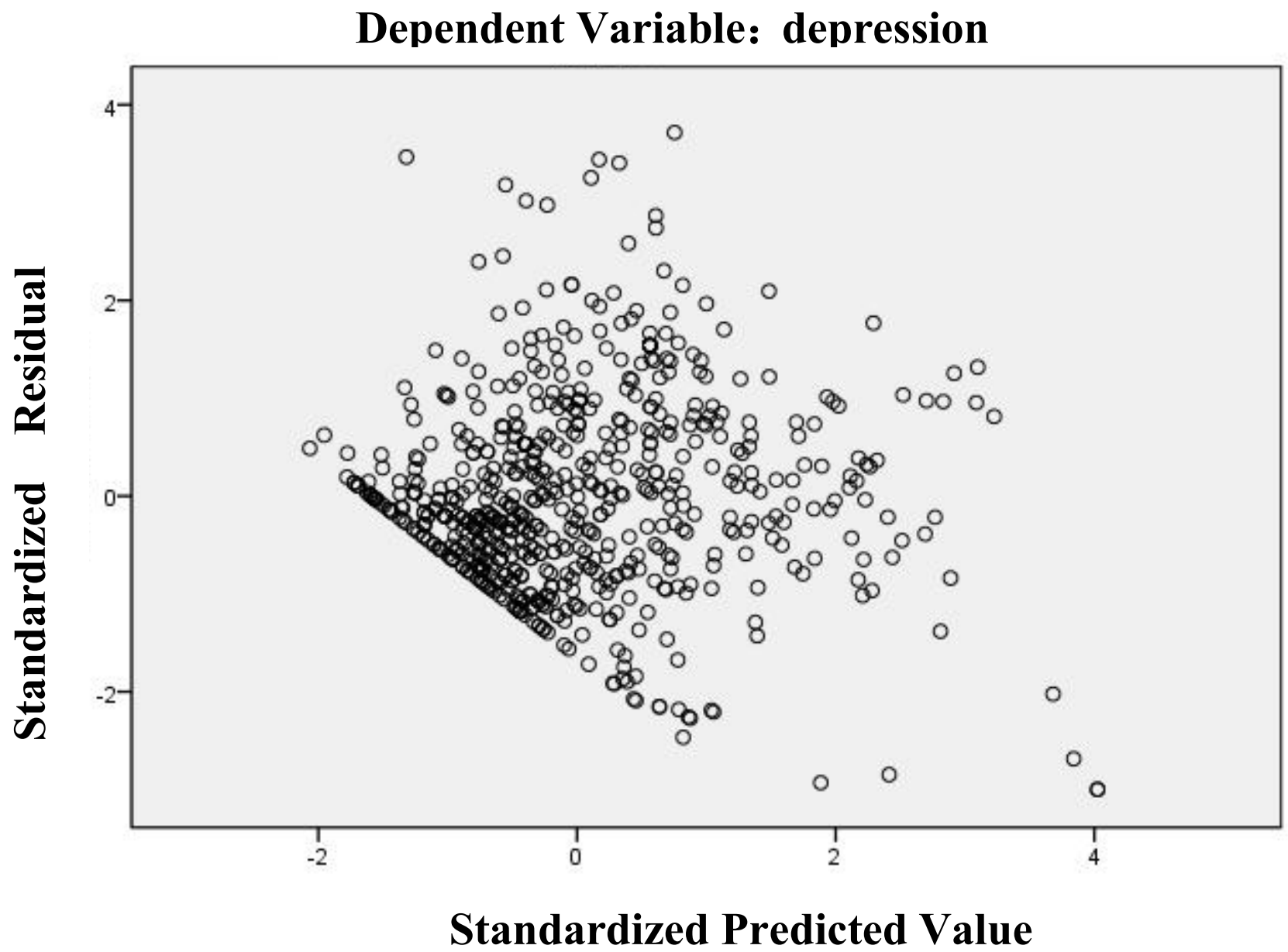

Table 2-3

Regression Coefficients and Multicollinearity Diagnostics

Variable	Unstandardized Coefficients		standardized Coefficients			Collinearity Statistics	
	b	*SE*	*β*	*t*	*Sig.*	*Tolerance*	*VIF*
Constant	4.669	2.379		1.962	0.050		
gender	1.172	0.635	0.051	1.845	0.066	0.974	1.027
El	-1.332	0.949	-0.039	-1.403	0.161	0.977	1.023
Bp	0.241	0.693	0.011	0.348	0.728	0.813	1.230
Fe	-0.509	0.625	-0.036	-0.814	0.416	0.377	2.653
Me	0.808	0.625	0.058	1.292	0.197	0.372	2.691
IPV	0.672	0.043	0.470	15.729	＜0.001	0.831	1.203
Brooding	1.297	0.148	0.363	8.790	＜0.001	0.436	2.292
refection	0.135	0.143	0.038	0.941	0.347	0.452	2.212

3.4 Equation 4: IPV+brooding+resilience+brooding×resilience + covariates→ depression

- **scatter plot**

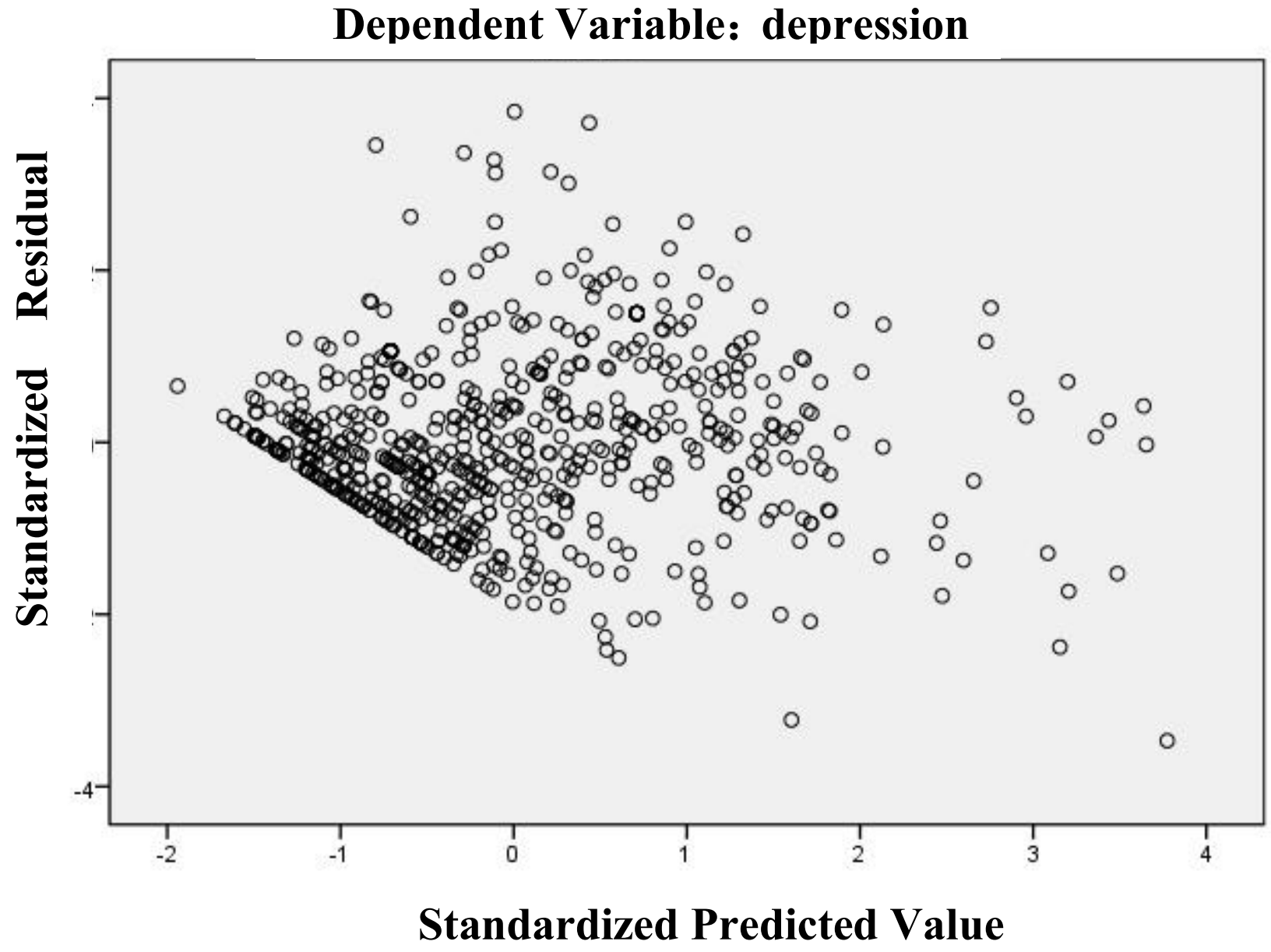

Table 2-4

Regression Coefficients and Multicollinearity Diagnostics

Variable	Unstandardized Coefficients		standardized Coefficients			Collinearity Statistics	
	b	*SE*	β	*t*	*Sig.*	*Tolerance*	*VIF*
Constant	34.625	2.992		11.572	<0.001		
gender	-0.059	0.562	-0.003	-0.105	0.916	0.950	1.053
El	-1.854	0.832	-0.054	-2.229	0.026	0.972	1.029
Bp	0.587	0.606	0.026	0.969	0.333	0.812	1.232
Fe	-0.170	0.546	-0.012	-0.311	0.756	0.377	2.654
Me	0.609	0.547	0.044	1.113	0.266	0.371	2.696
IPV	0.385	0.042	0.269	9.081	<0.001	0.646	1.549
Brooding	1.249	0.094	0.349	13.324	<0.001	0.825	1.212
resilience	-0.248	0.018	-0.387	-13.405	<0.001	0.680	1.470
*BRO*REF*	-1.084	0.242	-0.109	-4.471	<0.001	.959	1.043

4 Johnson–Neyman Analysis of the Moderated Mediation Effect

We further examined the moderating effect of resilience using the Johnson–Neyman method. Specifically, the analysis was conducted using the **PROCESS v4.0** macro in **SPSS Statistics 26.0**, with resilience specified as the moderator in the moderated mediation model. The Johnson–Neyman analysis provided by **PROCESS** was used to examine how the conditional effect of rumination on depressive symptoms varied across different levels of resilience. The results showed that there wa**s no statistically significant** transition point within the observed range of resilience, indicating that the conditional effect of rumination on depressive symptoms did not change from significant to non-significant, or vice versa, within the observed range. Although the conditional effect of rumination on depressive symptoms gradually weakened as resilience increased, the effect remained statistically significant throughout the observed range. This finding is consistent with the significant interaction between rumination and resilience.

5 Sensitivity Analysis of the Results Without Covariate Adjustment

5.1 Analyses of Linearity, Multicollinearity, and Homoscedasticity

5.1.1 Equation 1: IPV → brooding

- **scatter plot**

Dependent Variable：brooding

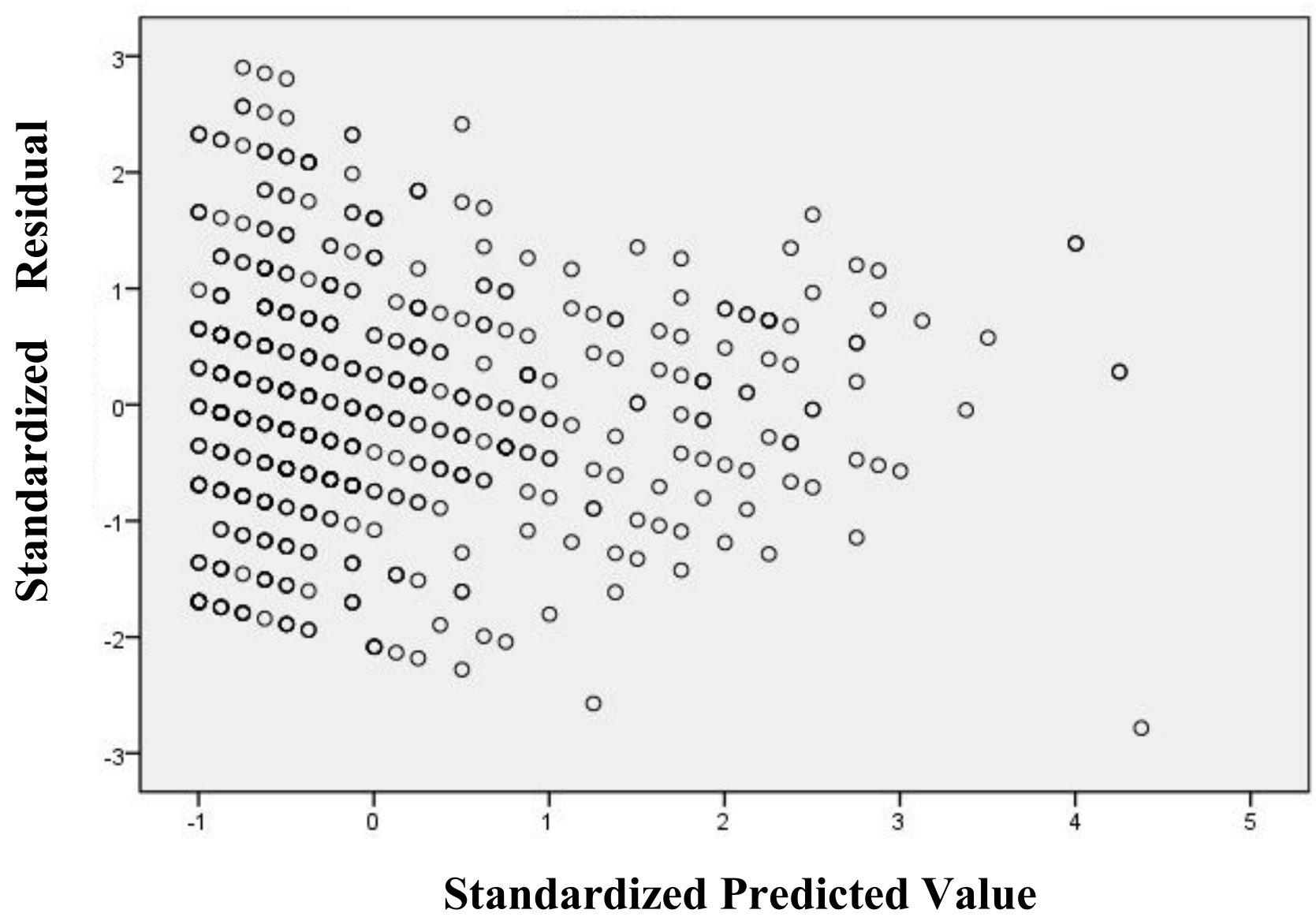

Table 3-1

Regression Coefficients and Multicollinearity Diagnostics

Variable	Unstandardized Coefficients		standardized Coefficients			Collinearity Statistics	
	b	*SE*	*β*	*t*	*Sig.*	*Tolerance*	*VIF*
Constant	8.60	0.29		30.15	<0.001		
IPV	0.15	0.01	0.36	10.02	<0.001	1.000	1.000

5.1.2 Equation 2: IPV → reflection

- **scatter plot**

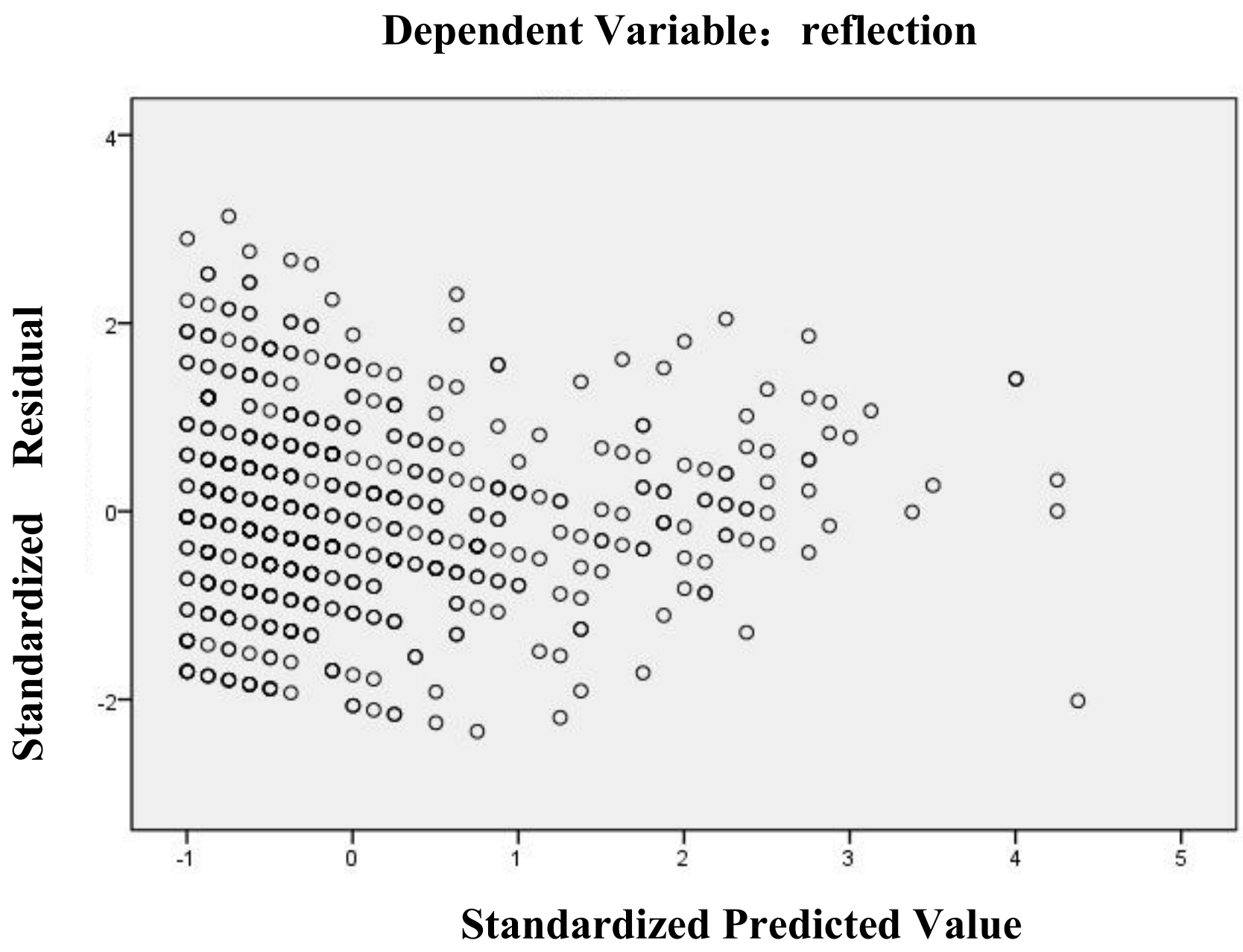

Table 3-2

Regression Coefficients and Multicollinearity Diagnostics

Variable	Unstandardized Coefficients		standardized Coefficients			Collinearity Statistics	
	b	*SE*	*β*	*t*	*Sig.*	*Tolerance*	*VIF*
Constant	8.80	0.30		30.22	<0.001		
IPV	0.14	0.02	0.34	9.35	<0.001	1.00	1.00

5.1.3 Equation 3: IPV + Brooding + Reflection→ depression

- **scatter plot**

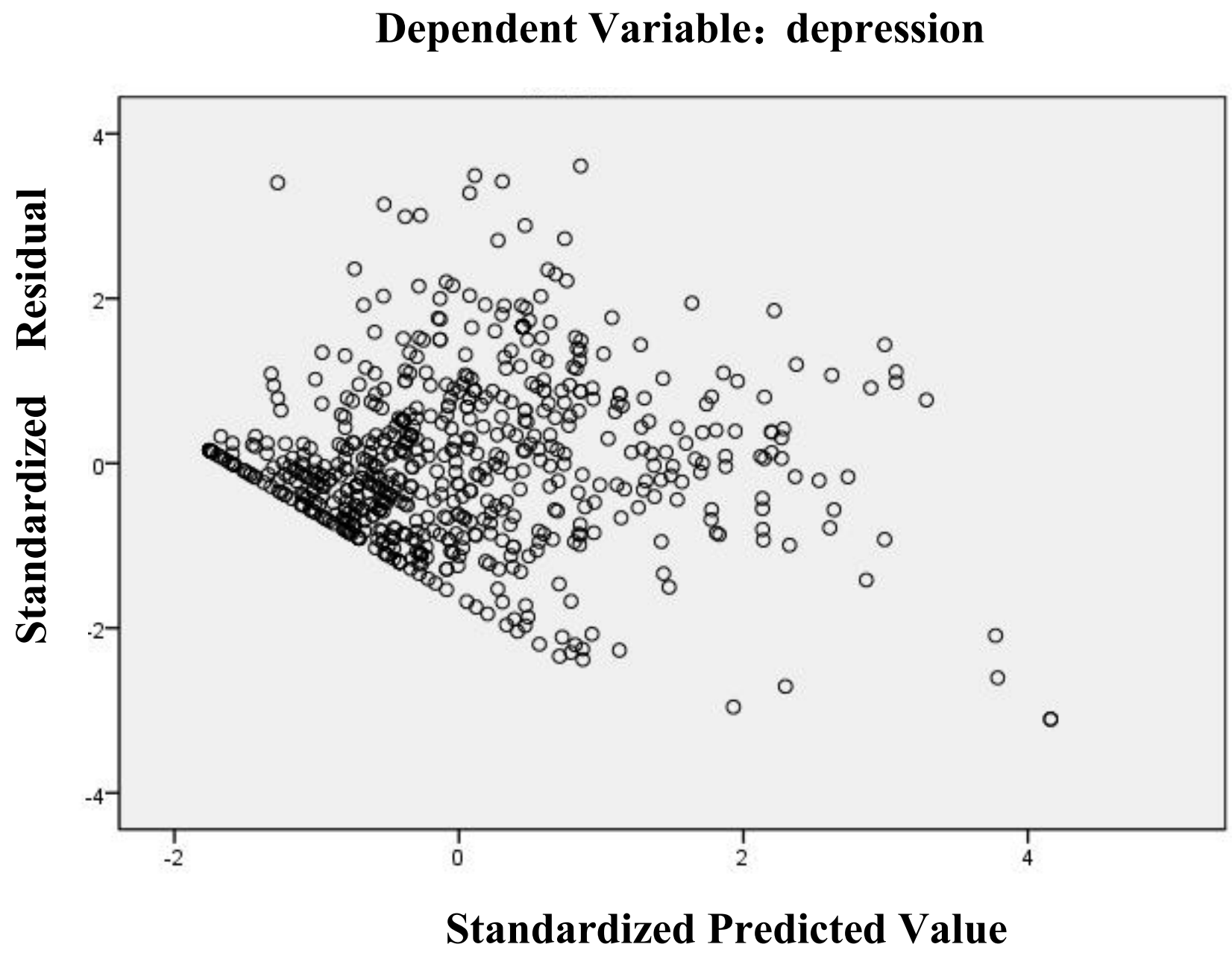

Table 3-3

Regression Coefficients and Multicollinearity Diagnostics

Variable	Unstandardized Coefficients		standardized Coefficients			Collinearity Statistics	
	b	*SE*	*β*	*t*	*Sig.*	*Tolerance*	*VIF*
Constant	5.90	1.25		4.70	＜0.001		
IPV	0.66	0.04	0.46	15.65	＜0.001	0.86	1.17
brooding	1.33	0.15	0.37	9.10	＜0.001	0.45	2.24
reflection	0.12	0.14	0.03	0.85	0.40	0.45	2.20

5.1.4 Equation 4:IPV+brooding+resilience+brooding×resilience→ depression

Dependent Variable：depression

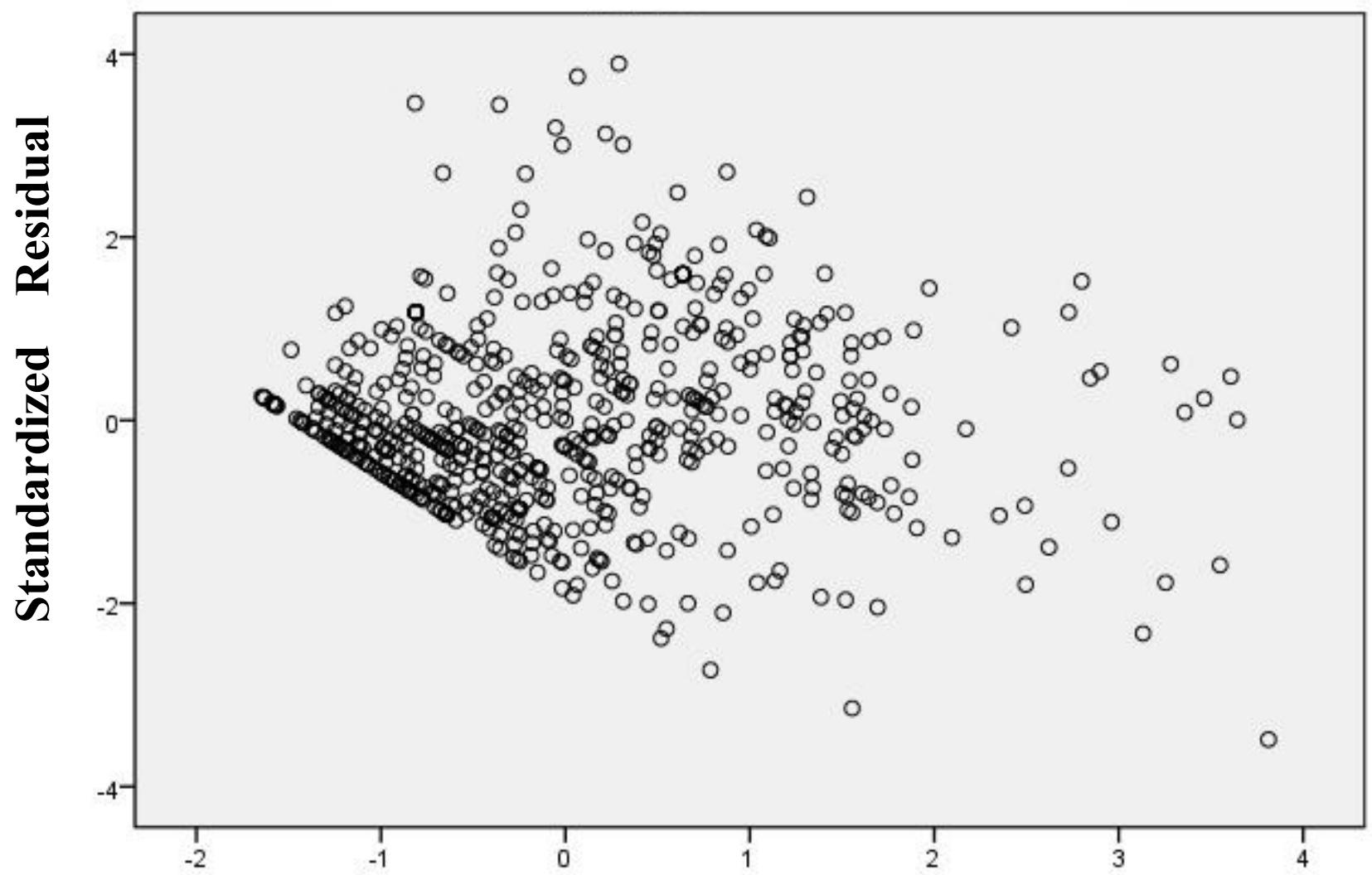

Standardized Predicted Value

Table 3-4

Regression Coefficients and Multicollinearity Diagnostics

Variable	Unstandardized Coefficients		standardized Coefficients			Collinearity Statistics	
	b	*SE*	*β*	*t*	*Sig.*	*Tolerance*	*VIF*
Constant	33.80	2.33		14.53	＜0.001		
IPV	0.37	0.04	0.261	8.87	＜0.001	0.66	1.52
brooding	1.27	0.09	0.354	13.66	＜0.001	0.85	1.18
resilience	-0.24	0.02	-0.380	-13.28	＜0.001	0.70	1.43
*BRO*REF*	-1.14	0.24	-0.114	-4.72	＜0.001	0.98	1.02

5.2 Parallel Mediation Effects of Different Rumination Subtypes

Path	*β*	*Effect*	*BootSE*	*t*	*p*	*BootLLCI*	*BootULCI*	R^2
**Brooding**								0.13
IPV→Brooding	0.36		0.04	10.02	< .001	0.29	0.43	
**Reflection**								0.12
IPV→Reflection	0.34		0.04	9.35	< .001	0.27	0.41	
**Depression**								0.51
IPV→depression	0.46		0.04	15.65	< .001	0.40	0.52	
Brooding→depression	0.37		0.05	9.10	< .001	0.28	0.46	
Resilience→depression	0.03		0.04	0.85	0.40	−0.05	0.11	
**Total effect**		0.61	0.03	19.75	< .001	0.55	0.67	

**IPV→BRO→DEP**	0.14	0.02	0.10	0.18
**IPV→REF→DEP**	0.01	0.01	−0.02	0.04
**Total indirect**	0.15	0.02	0.11	0.18

5.3 Moderated Effects of Resilience

Path	β	*Effect*	*BootSE*	*t*	*p*	*BootLLCI*	*BootULCI*	R^2	ΔR^2
**Brooding**								0.13	
IPV→Brooding	0.36		0.04	10.02	< .001	0.29	0.43		
**Depression**								0.63	
IPV→depression	0.26		0.04	8.87	< .001	0.19	0.33		
Brooding→depression	0.35		0.03	13.66	< .001	0.30	0.40		
Resilience→depression	−0.38		0.03	−13.28	< .001	−0.44	−0.32		
**Brooding×Resilience →depression**	−0.10		0.02	−4.72	< .001	−0.14	−0.06		0.01
**IPV→BRO→DEP**									
**Low** resilience **(-1 SD)**		0.16	0.02			0.12	0.21		
**Mean** resilience		0.13	0.02			0.10	0.16		
**High** resilience **(+1 SD)**		0.09	0.01			0.07	0.12		
**Index moderated mediation**		−0.04	0.01			−0.05	-0.02		
